# Supplementary material for: The Status of Molecular Analyses of Isolates of Acanthamoeba Maintained by International Culture Collections
Source: Microorganisms. 2023 Jan 23;11(2):295. doi: 10.3390/microorganisms11020295 (PMC9961329; doi:10.3390/microorganisms11020295)
Supplement: Supplementary file 1 [file microorganisms-11-00295-s001.zip › Table S9 - sequence references.pdf]

microorganisms

Supplementary Table S9. References for sources of DNA sequence information for cultures of *Acanthamoeba* from ATCC, CCAP or BEI.

The table includes information on sequence accession number from GenBank and associated source references for isolates listed in Appendices Tables A1-A8.

1. References for DNA sequence sources of cultures that are currently available from ATCC, with equivalent CCAP or BEI identifiers

ATCC 30010 (CCAP 1501/1A)	[39,45,83–85,87–91]
ATCC 30011 (CCAP 1501/10)	[89,93]
ATCC 30134	[27,94]
ATCC 30135 (CCAP 1501/5)	[26,27,41]
ATCC 30137 (CCAP 1534/1)	[26,27,41,95]
ATCC 30171	[26,95,96]
ATCC 30173	[57]
ATCC 30234	[41,47,97]
ATCC 30461	[98,99,100]
ATCC 30487	[26,103]
ATCC 30730	[26]
ATCC 30731 (CCAP 1501/4)	[27,28,41]
ATCC 30732	[59,101]
ATCC 30841	[27,95,102]
ATCC 30866	[26,27]
ATCC 30867	[26,27]
ATCC 30868 (CCAP 1501/2G)	[103,105]
ATCC 30869 (CCAP 1534/3)	[89]
ATCC 30870 (CCAP 1547/1)	[27,41,89,95,103,105]
ATCC 30871 (CCAP 1501/3A)	[26,41,103]
ATCC 30872 (CCAP 1501/3B)	[98]
ATCC 30873 (CCAP 1501/3D)	[8,26,27]
ATCC 30884 (CCAP 1501/7)	[27,28,41]
ATCC 30901 (CCAP 1501/9)	[98,105]
ATCC 30921	[27]
ATCC 30973	[95,99,106]
ATCC 50238	[70,106]
ATCC 50239	[70]
ATCC 50240	[95,107]
ATCC 50241	[70,95]
ATCC 50243	[34]
ATCC 50251	[106]
ATCC 50252	[8,26]
ATCC 50253	[95,106,108]
ATCC 50254	[17,40,89,95,106]
ATCC 50368	[27,28]
ATCC 50369	[27,28]
ATCC 50370	[8,27,28,37,95]
ATCC 50371	[27,28]
ATCC 50372	[27,28,37,95]
ATCC 50373 (CCAP 1501/1B)	[27,28,37]
ATCC 50374	[27,28]
ATCC 50388	[26]
ATCC 50427	[103]
ATCC 50428	[103]

ATCC 50429	[103]
ATCC 50435	[26]
ATCC 50436	[38]
ATCC 50491	[27,28]
ATCC 50492	[27,28,111]
ATCC 50493	[27,28,112]
ATCC 50494	[27,28]
ATCC 50495 (NR-46462)	[27,28]
ATCC 50496	[27,28,95,113]
ATCC 50497	[27,28]
ATCC 50498	[27,28]
ATCC 50662	[38]
ATCC 50664	[38]
ATCC 50665	[38]
ATCC 50669	[38]
ATCC 50670	[38]
ATCC 50675	[38]
ATCC 50676	[27,40]
ATCC 50677	[27,40]
ATCC 50678	[27,40]
ATCC 50679	[27,40]
ATCC 50680	[27,40]
ATCC 50681	[27,40]
ATCC 50682	[27,40]
ATCC 50683	[27,40]
ATCC 50684	[27,40]
ATCC 50685	[27,40]
ATCC 50686	[27,40]
ATCC 50687	[27,40]
ATCC 50690	[103]
ATCC 50691	[103]
ATCC 50692	[103]
ATCC 50702	[114]
ATCC 50703	[97,103]
ATCC 50704 (NR-10462)	[103,115]
ATCC 50705	[103]
ATCC 50706	[97,103]
ATCC 50707	[97,103]
ATCC 50708 (NOT CCAP 1501/3c)	[26]
ATCC 50709	[26,27]
ATCC 50710	[26]
ATCC 50711	[26]
ATCC 50722	[112]
ATCC 50727	[26]
ATCC 50739	[4,97]
ATCC PRA-1	[116]
ATCC PRA-2	[38]
ATCC PRA-3	[57]
ATCC PRA-4	[116]
ATCC PRA-7	[116]
ATCC PRA-8	[116]
ATCC PRA-9	[57]
ATCC PRA-10	[116]
ATCC PRA-11	[116]

ATCC PRA-12	[38]
ATCC PRA-75 (NR-33654)	[70]
ATCC PRA-76 (NR-33655)	[70]
ATCC PRA-77 (NR-33656)	[70]
ATCC PRA-78 (NR-33657)	[70]
ATCC PRA-79 (NR-33658)	[70]
ATCC PRA-80 (NR-33659)	[70]
ATCC PRA-81 (NR-33660)	[70]
ATCC PRA-82 (NR-33661)	[70]
ATCC PRA-83 (NR-33662)	[70]
ATCC PRA-84 (NR-33663)	[70]
ATCC PRA-105	[117]
ATCC PRA-106	[118]
ATCC PRA-107	[117]
ATCC PRA-108	[119]
ATCC PRA-112 (NR-10461)	[119]
ATCC PRA-113	[117]
ATCC PRA-114	[117]
ATCC PRA-115	[119]
ATCC PRA-219	[116]
ATCC PRA-220	[120]
ATCC PRA-221	[72]
ATCC PRA-222	[72]
ATCC PRA-223	[72]
ATCC PRA-225	[72]
ATCC PRA-226	[72]
ATCC PRA-227	[72]
ATCC PRA-228	[72]
ATCC PRA-287	[115,122]
ATCC PRA-411 (NR-46481)	[54]

2. References for DNA sequence sources of cultures that are currently available from CCAP and that are not equivalent to any ATCC culture

CCAP 1501/3c (NOT ATCC 50708	[26,27,41]
CCAP 1501/8	[89]
CCAP 1501/14	[89]
CCAP 1501/17	[89]
CCAP 1501/18	[123,73]
CCAP 1501/19	[56]
CCAP 1534/2	[89]

3. References for DNA sequence sources of cultures that are currently available from BEI and that are not equivalent to any ATCC culture

NR-46463 (CDC:V036)	[112]
NR-46466 (CDC:V313)	[70]
NR-46467 (CDC:V329)	[70]
NR-46468 (CDC:V333)	[112]
NR-46470 (CDC:V411)	[70]
NR-46473 (CDC:V522)	[70]
NR-46476 (CDC:V548)	[125]

4. References for DNA sequence sources of cultures that are currently available from BEI but that are equivalent to inactive ATCC cultures

NR-46460 (ATCC 50656)	[70]
NR-46461 (ATCC 50657)	[70]

5. References for DNA sequence sources of inactive cultures or cultures no longer available from culture centers

ATCC 50659	[70]
ATCC 50723	[26]
ATCC 50724	[26,27,109]
ATCC 50725	[26]
ATCC 50726	[126]
ATCC PRA-224	[121]
